# Supplementary figures and images for: Spatiotemporal Regulation of De Novo and Salvage Purine Synthesis during Brain Development
Source: eNeuro. 2023 Oct 9;10(10):ENEURO.0159-23.2023. doi: 10.1523/ENEURO.0159-23.2023 (PMC10566546; doi:10.1523/ENEURO.0159-23.2023)

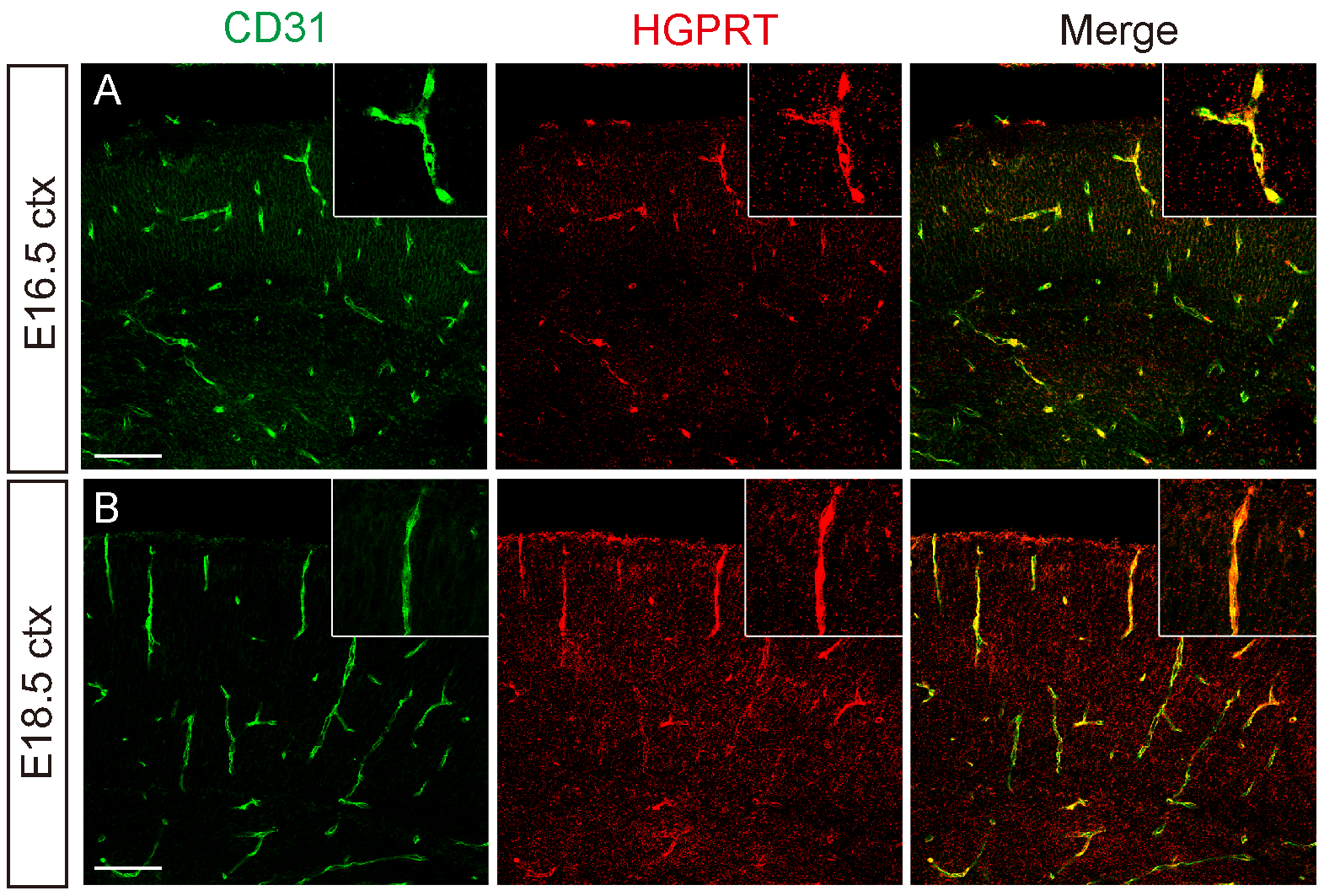

Supplement: Extended Data Figure 3-1 — HGPRT is expressed in CD31+ endothelial cells in the developing cerebral cortex. E16.5 (A) and E18.5 (B) cerebral cortices double immunostained with anti-CD31 (green) and anti-HGPRT (red) antibodies. Insets present magnified views of each blood vessel. Scale bar, 50 μm. Download Figure 3-1, TIF file. [file enu-eN-NWR-0159-23-s02.tif]

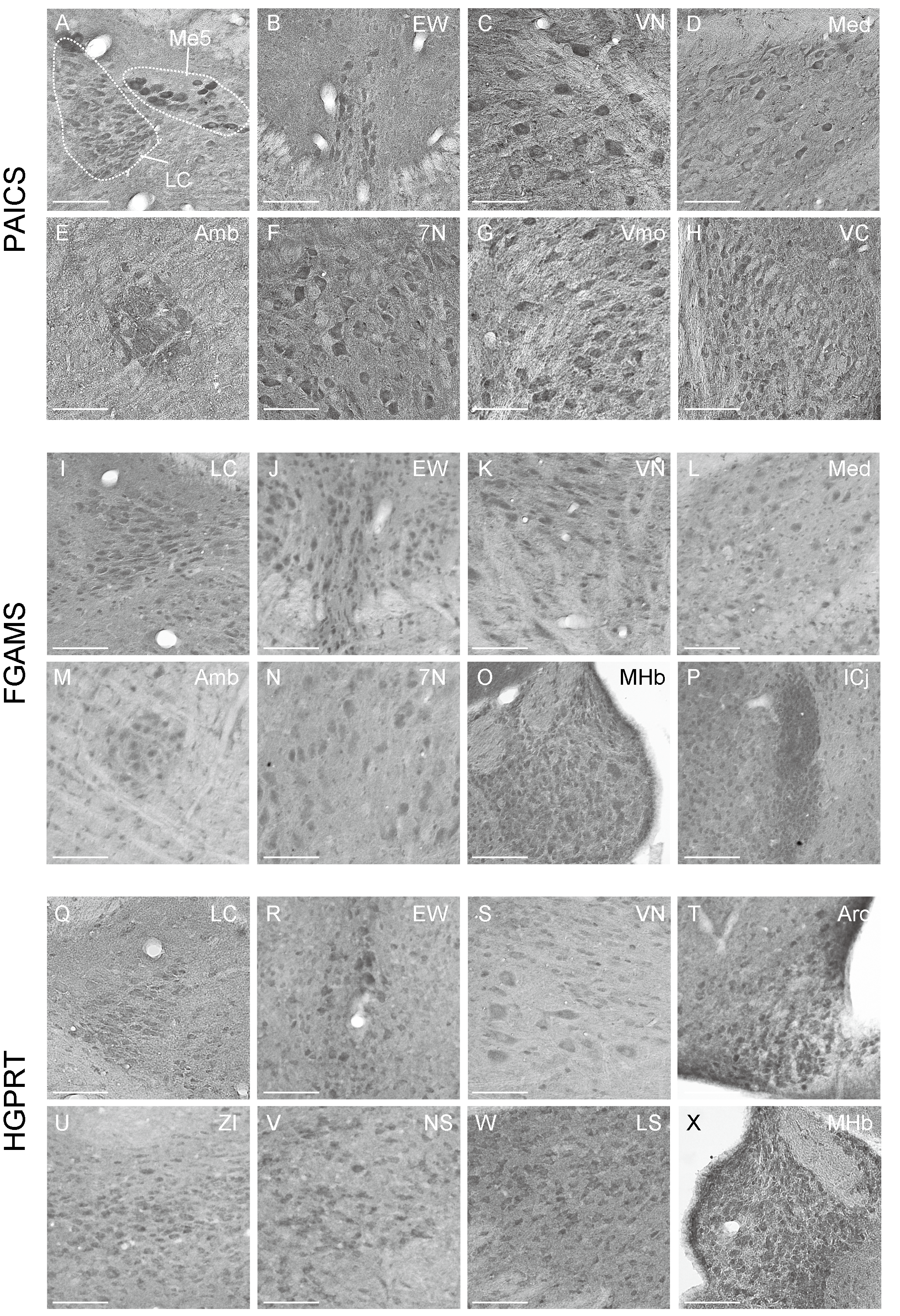

Supplement: Extended Data Figure 3-2 — Expression of purine synthesis enzymes in the adult brain. A–X, Coronal sections of adult brains immunostained with anti-PAICS (A–H), anti-FGAMS (I–P), and anti-HGPRT (Q–X) antibodies. Representative images of the locus coeruleus (LC; A, I, Q), Edinger–Westphal nucleus (EW; B, J, R), vestibular nucleus (VN; C, K, S), medial vestibular nucleus (Med; D, L), ambiguous nucleus (Amb; E, M), facial nucleus (7N; F, N), trigeminal motor nucleus (Ⅴmo; G), ventral cochlear nucleus (VC; H), medial habenular nucleus (MHb; O, X), islands of Calleja (ICj; P), arcuate hypothalamic nucleus (Arc; T), zona incerta (ZI; U), nigrostriatal bundle (NS; V), and lateral septal (LS; W). Areas surrounded by a dashed line in A denote the LC and mesencephalic trigeminal nucleus (Me5). Scale bar, 20 μm. Download Figure 3-2, TIF file. [file enu-eN-NWR-0159-23-s03.tif]

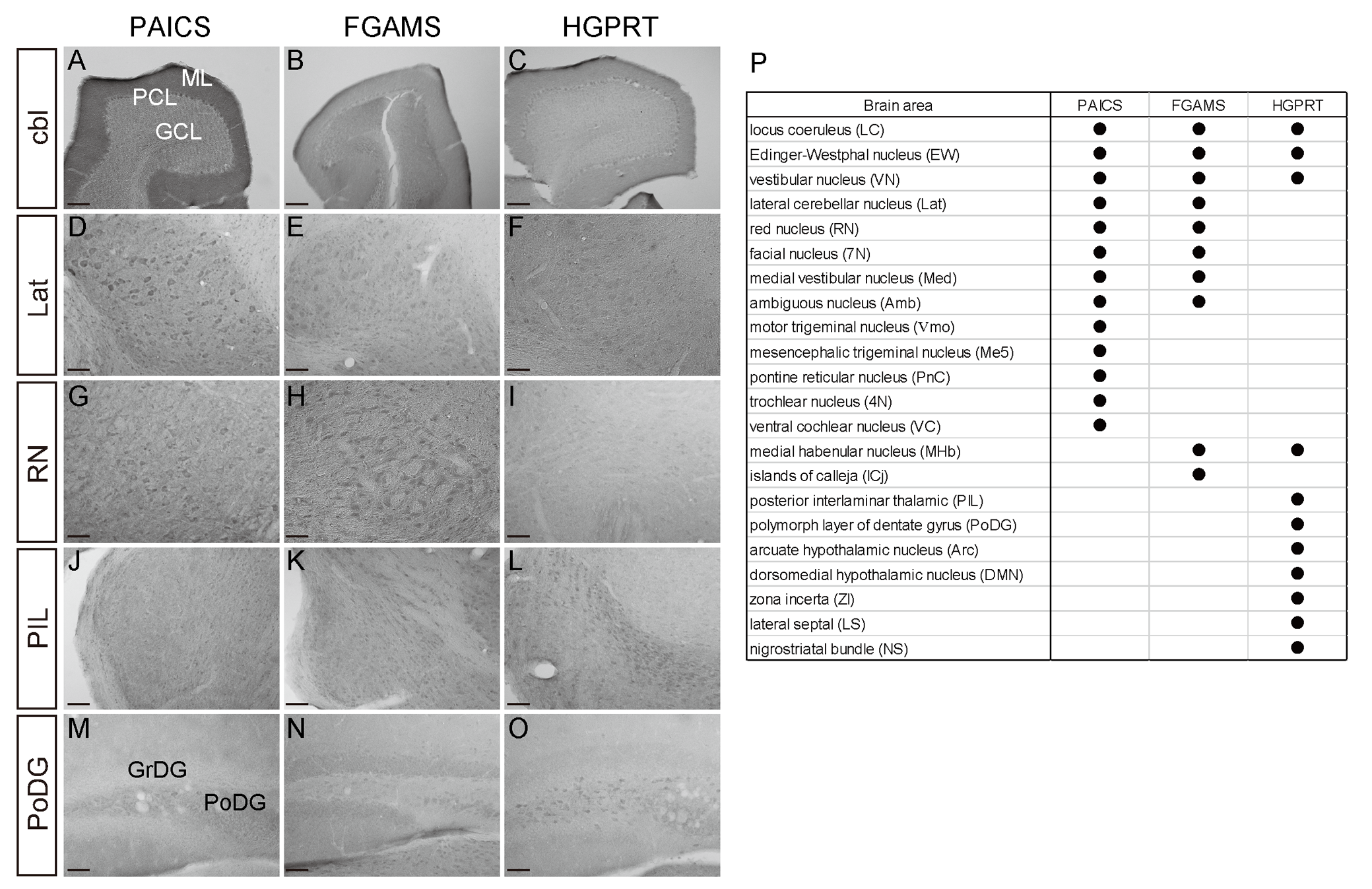

Supplement: Extended Data Figure 3-3 — Expression of purine synthesis enzymes in the adult brain. A–P, Coronal sections of adult brains immunostained with anti-PAICS (A, D, G, J, M), anti-FGAMS (B, E, H, K, N), and anti-HGPRT (C, F, I, L, O) antibodies. Representative images of the cerebellum (cbl; A–C), lateral cerebellar nucleus (Lat; D–F), red nucleus (RN; G–I), posterior interlaminar thalamic (PIL) complex (J–L), and polymorph layer of the dentate gyrus (PoDG; M–O). P, Expression profiles of PAICS, FGAMS, and HGPRT in discrete brain regions and nuclei. Black circles indicate regions with high immunoreactivity. ML, molecular cell layer; PCL, Purkinje cell layer; GCL, granule cell layer; GrDG, granule cell layer of the dentate gyrus. Scale bar, 100 μm (A–O). Download Figure 3-3, TIF file. [file enu-eN-NWR-0159-23-s04.tif]

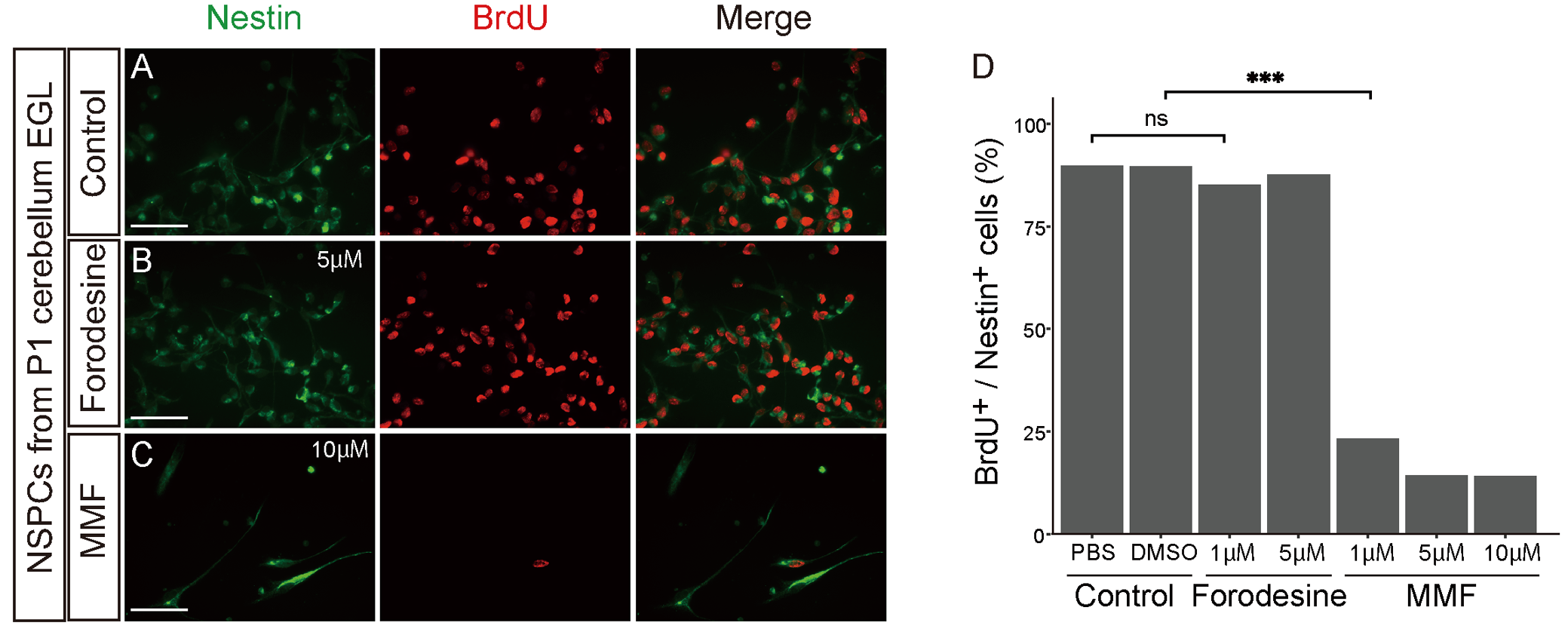

Supplement: Extended Data Figure 4-1 — Inhibition of de novo purine synthesis disturbs the proliferation of cerebellar NSPCs in vitro. Primary cultured NSPCs derived from P1 EGL were treated for 48 h with PBS or DMSO (control; A), forodesine (1 and 5 μm; B), or MMF (1, 5, and 10 μm; C), followed by BrdU labeling for 24 h. NSPCs were immunostained with anti-Nestin (green) and anti-BrdU (red) antibodies. D, Quantification of dividing NSPCs. The ratio of the number of BrdU+ Nestin+ cells to the total number of Nestin+ cells. ns, not significant; ***p < 0.001, χ2 test with Holm–Bonferroni correction. Control PBS, n = 460; control DMSO, n = 396; forodesine 1 μm, n = 373; forodesine 5 μm, n = 700; MMF 1 μm, n = 73; MMF 5 μm, n = 70; MMF 10 μm, n = 64. Scale bar, 50 μm. Download Figure 4-1, TIF file. [file enu-eN-NWR-0159-23-s05.tif]

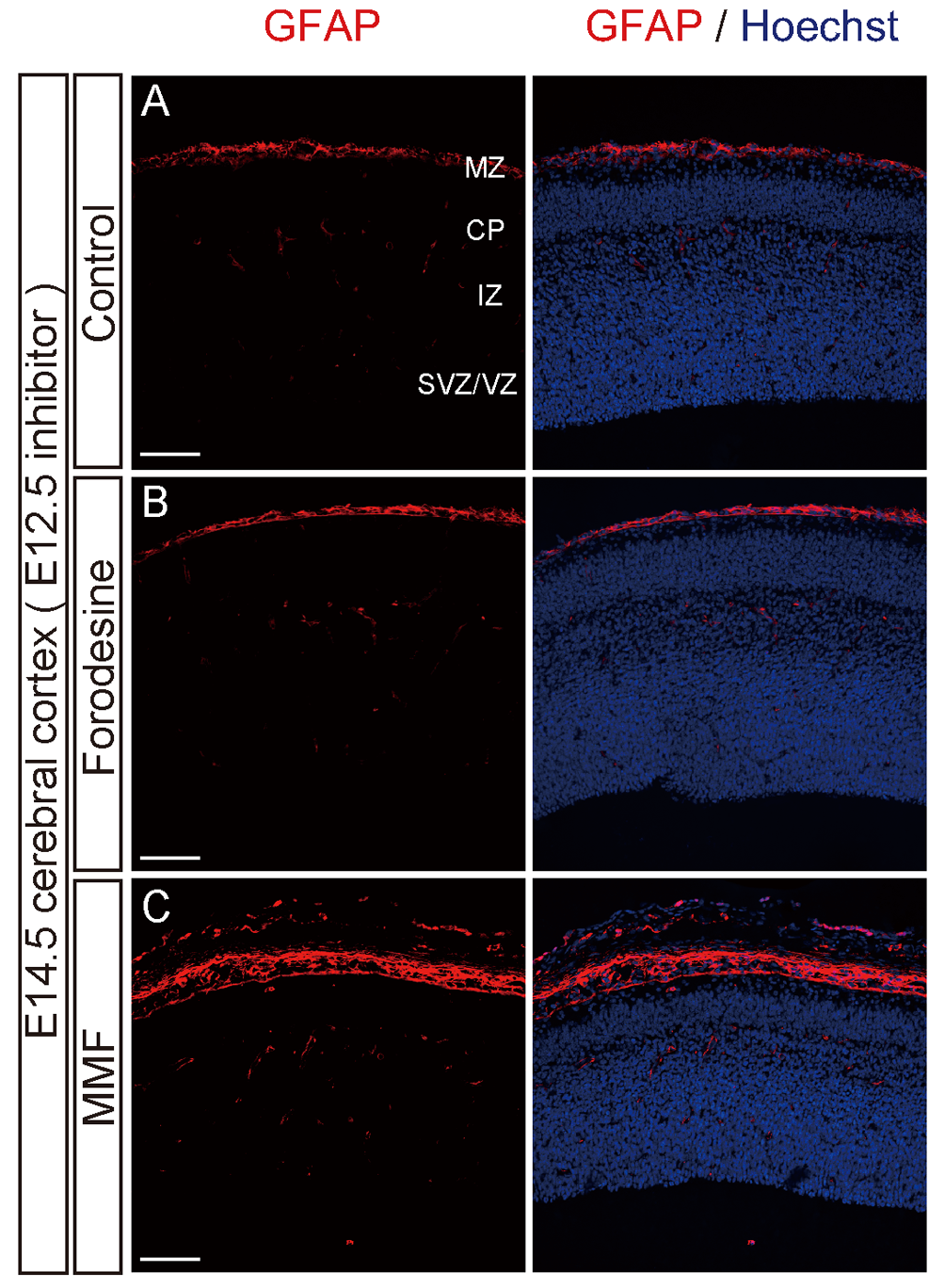

Supplement: Extended Data Figure 6-1 — Expression of GFAP in cerebral cortices treated with purine synthesis inhibitors. E12.5 embryos were treated with control DMSO (A), forodesine (B), or MMF (C) and analyzed at E14.5. Horizontal frozen sections were immunostained with anti-GFAP antibody (red). Nuclei were stained with Hoechst dye (blue). MZ, marginal zone; CP, cortical plate; IZ, intermediate zone; SVZ/VZ, subventricular zone/ventricular zone. Scale bar, 100 μm (A–F). Download Figure 6-1, TIF file. [file enu-eN-NWR-0159-23-s06.tif]

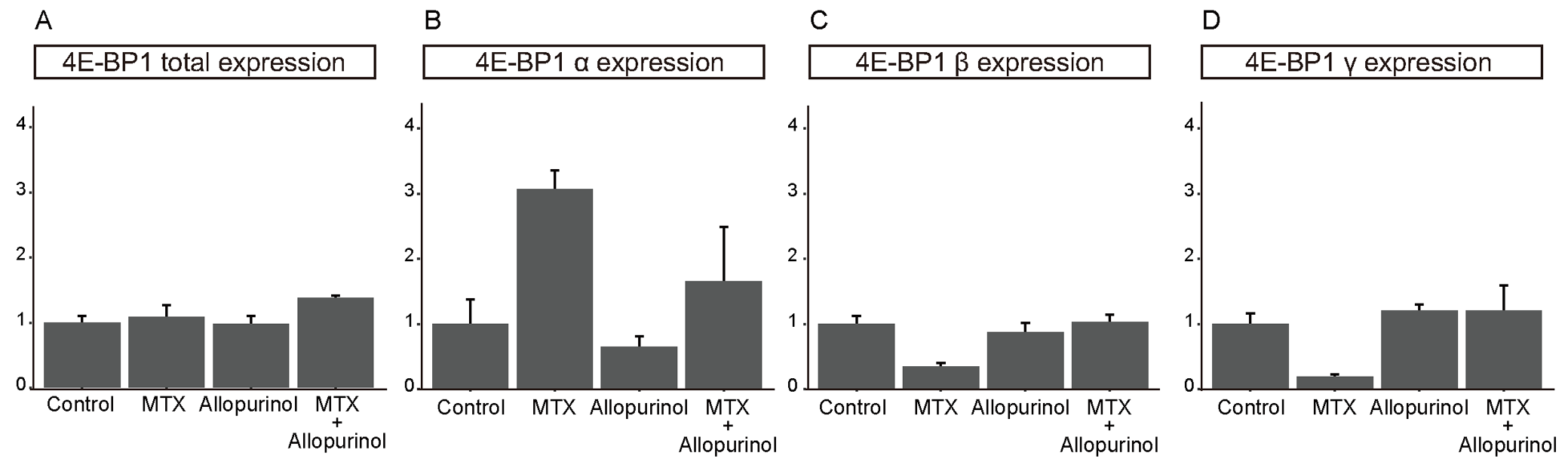

Supplement: Extended Data Figure 7-1 — 4E-BP1 expression. Quantified comparison of the total protein content of 4E-BP1 (A) and each isoform [4E-BP1-α (B), 4E-BP1-β (C), or 4E-BP1-γ (D)] in Figure 7A. Protein bands obtained using three independent mouse brains treated with each drug (DMSO, MTX, allopurinol, or MTX and allopurinol) were quantified using α-Tubulin as an internal standard. Data are presented as the mean ± SEM. Download Figure 7-1, TIF file. [file enu-eN-NWR-0159-23-s07.tif]

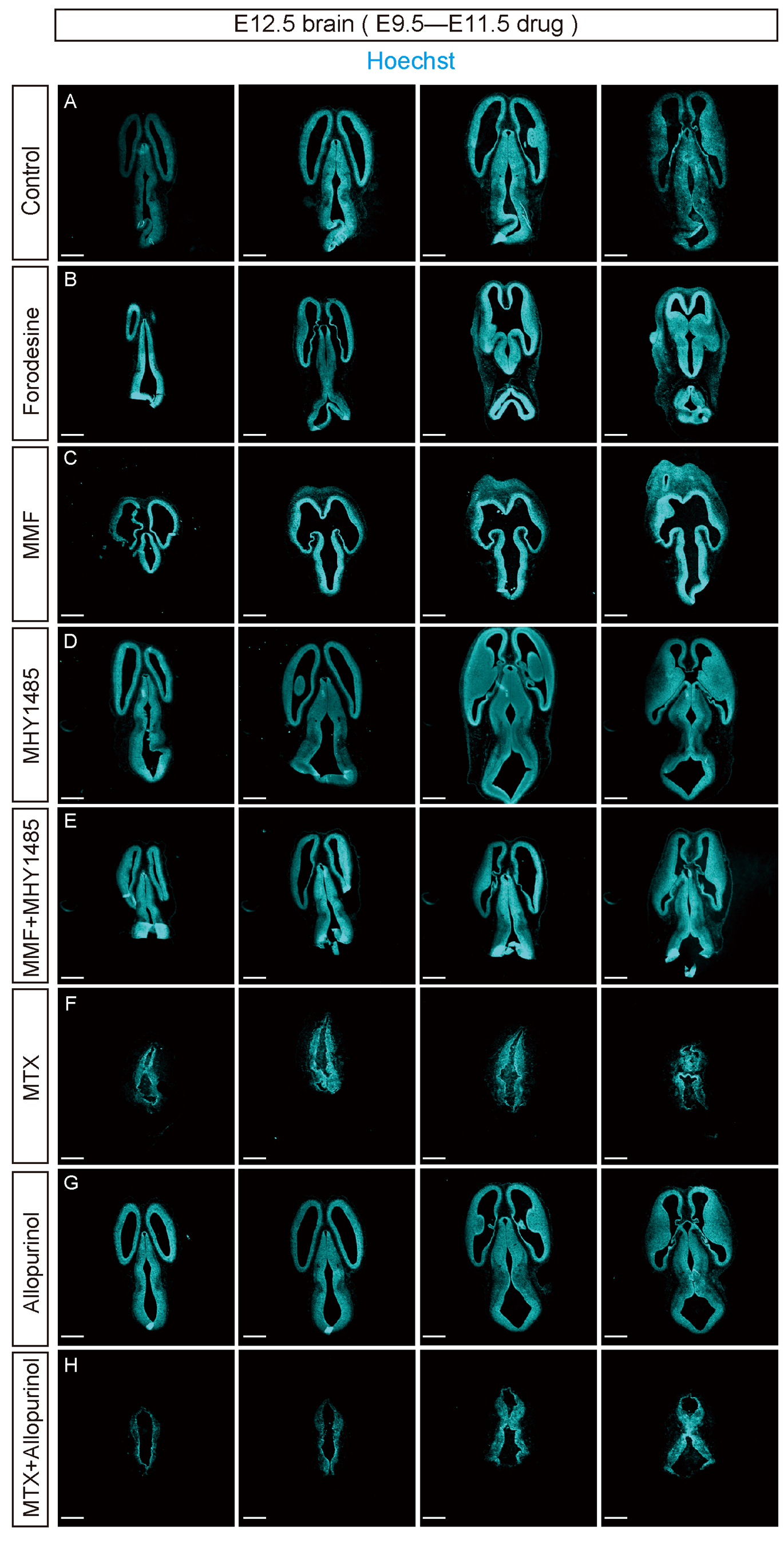

Supplement: Extended Data Figure 9-1 — Brain malformations caused by the inhibition of the de novo pathway. Serial horizontal sections of each E12.5 embryo were treated with different inhibitors. The left and right panels represent the sections cutting through the dorsal and ventral planes, respectively. The pregnant mice were successively treated with control DMSO (A), forodesine (B), MMF (C), MHY1485 (D), MMF and MHY1485 (E), MTX (F), allopurinol (G), or MTX and allopurinol (H) during E9.5–E11.5, and the brains were harvested at E12.5. Sections were stained with Hoechst dye (cyan). Scale bar, 500 μm. Download Figure 9-1, TIF file. [file enu-eN-NWR-0159-23-s08.tif]

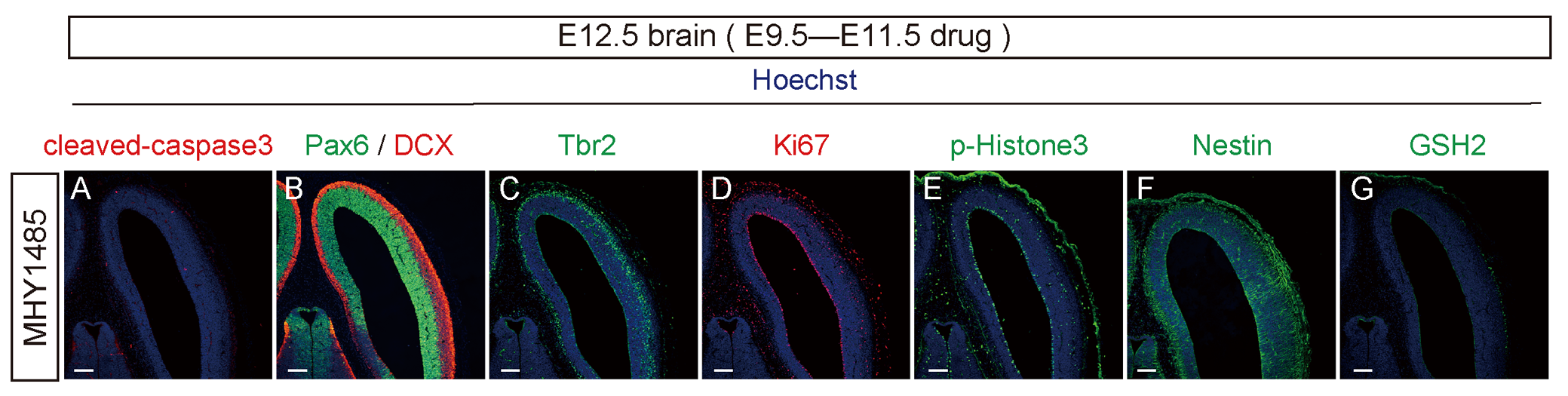

Supplement: Extended Data Figure 10-1 — Embryonic brains treated alone with the mTOR activator MHY1485. MHY1485 was administered to pregnant mice between E9.5 and E11.5, followed by analysis at E12.5. Horizontal frozen sections were immunostained with antibodies against cleaved-caspase3 (red; A), Pax6 (green)/DCX (red; B), Tbr2 (green; C), Ki67 (red; D), pH3 (green; E), Nestin (green; F), or GSH2 (green; G). Scale bar, 100 μm (A–G). Download Figure 10-1, TIf file. [file enu-eN-NWR-0159-23-s09.tif]
